# A Hopeful Natural Product, Pristimerin, Induces Apoptosis, Cell Cycle Arrest, and Autophagy in Esophageal Cancer Cells

**DOI:** 10.1155/2019/6127169

**Published:** 2019-05-14

**Authors:** Peng Huang, Li-Ying Sun, Yan-Qiao Zhang

**Affiliations:** ^1^Department of Gastrointestinal Oncology, Harbin Medical University Cancer Hospital, Harbin 150081, China; ^2^Department of Gastroenterology, The First Affiliated Hospital of Harbin Medical University, Harbin 150081, China

## Abstract

Esophageal cancer is one of the most common malignant digestive diseases worldwide. Although many approaches have been established for the treatment of esophageal cancer, the survival outcome has not improved. Pristimerin is a quinone methide triterpenoid with anticancer, antiangiogenic, anti-inflammatory, and antiprotozoal activities. However, the role of pristimerin in cancers such as esophageal cancer is unclear. In this study, we investigated the role and mechanisms of action of pristimerin in esophageal cancer. First, we found that pristimerin can induce apoptosis in esophageal cancer *in vivo* and *in vitro*. CCK-8 and clonogenic assays showed that pristimerin decreased the growth of Eca109 cells. In addition, we found that pristimerin decreased the protein expression of CDK2, CDK4, cyclin E, and BCL-2 and increased the expression of CDKN1B. Meanwhile, pristimerin elevated the ratio of LC3-II/LC3-I. Otherwise, downregulation of CDKN1B can reduce the esophageal cancer tumor growth induced by pristimerin. In conclusion, our findings revealed an important role of pristimerin in esophageal cancer and suggest that pristimerin might be a potential therapeutic agent for this cancer.

## 1. Introduction

Esophageal cancer (ESC) is one of the least studied and deadliest cancers worldwide, and it ranks sixth among all cancers in mortality [1]. Numerous epidemiologic studies show that smoking, hot tea drinking, alcohol consumption, and low intake of fresh fruit and vegetables are contributing risk factors for ESC. And the most important precancerous disease is Barrett's esophagus. The proportion of adenocarcinoma and squamous cell carcinoma is more than 95% in esophageal cancers [2, 3]. In China, esophageal squamous cell carcinoma is the predominant esophageal cancer, and it occurs at a rate 20 to 30 times higher than in the United States. Despite improvement therapies, it still has extremely aggressive nature and a poor survival rate [4, 5]. Future endeavors will need to focus on development of new chemotherapies to prolong the survival of patients with esophageal cancer.

Pristimerin (20*α*-3-hydroxy-2-oxo-24-nor-friedela-1-10,3,5,7-tetraen-carboxylic acid-29-methylester) is a natural quinonoid triterpene isolated from the plant families Celastraceous and Hippocratic. Pristimerin exhibits anticancer activity *in vitro* via suppression of cell cycle progression [6] and angiogenesis [7] and induction of apoptosis [8–11]. It has been previously reported that pristimerin inhibits lipopolysaccharide-induced production of inflammatory mediators in murine macrophages via downregulation of nuclear factor- (NF-) *κ*B and mitogen-activated protein kinase signal pathways [12].

In this study, we found that pristimerin reduced proliferation and growth and induced cell cycle arrest and apoptosis in Eca109 esophageal cancer cells. Additionally, we found that pristimerin induced autophagy in Eca109 cells. Our findings revealed an important role for pristimerin in esophageal cancer and suggest that pristimerin may be a potential therapeutic agent for patients with this cancer.

## 2. Materials and Methods

### 2.1. Cell Culture

The human esophageal cancer cell line Eca109 (Laboratory of Medical Genetics, Department of Biology, Harbin Medical University) was grown in RPMI 1640 medium containing 10% heat-inactivated fetal bovine serum and 100 units/mL penicillin/streptomycin. Cells were maintained in a humidified atmosphere of 5% CO_2_ at 37°C. After confluence, cells were subcultured using trypsin digestion. Cells in the logarithmic phase were selected for study. The methods used in our manuscript were followed as our previous work [13, 14].

### 2.2. Plasmid and siRNA Transfection

Cells were seeded into 35 mm plates 24 h prior to transfection. To achieve silencing, CDKN1B (Cat: stQ0001993-1, RiboBio, Guangzhou, China) was transfected with CDKN1B siRNA or control siRNA using lipofectamine 2000 (Invitrogen, Carlsbad, CA) with serum-free medium according to the manufacturer's instructions. Five hours after transfection, the medium was changed to complete medium, and cells were subsequently cultured for 48 hours.

### 2.3. Antibodies and Western Blotting

Cells were lysed with RIPA lysis buffer containing a protease inhibitor cocktail (Roche, Basel, Switzerland). Equal amounts of proteins were separated by SDS-PAGE and transferred to a nitrocellulose membrane (Pall Corporation, Port Washington, NY). After blocking, the blots were probed with primary antibodies against *β*-actin (1 : 2000), cyclin E (1 : 1000), Bcl-2 (1 : 1000) (Santa Cruz Biotechnology, Dallas, TX), CDK2 (1 : 1000), CDK4 (1 : 1000), and LC3-II/LC3-I (1 : 1000) (Cell Signaling Technology, USA). After washing and incubating with rabbit or mouse secondary antibodies (1 : 10000) (Cell Signaling Technology, Danvers, MA), the blots were visualized using the ECL reagent (GE Healthcare, Chicago, IL).

### 2.4. CCK-8 Cell Viability Assay

Cells transfected with CDKN1B siRNA, control siRNA, or pristimerin (0.5, 1.0, 1.5, 2.0, and 4.0 *μ*mol·L^–1^) were seeded into 96-well plates at a density of 2 × 10^3^ per well and cultured for 48 hours. Cell viability was assessed by Cell Counting Kit-8 (Dojindo, Tokyo, Japan).

### 2.5. Clonogenic Survival Assay

Next, 8 × 10^2^ cells transfected with CDKN1B siRNA, control siRNA, or pristimerin were counted and seeded in 6 cm dishes. After 10 days of culturing, colonies were stained with 0.1% crystal violet in 20% methanol for 15 min. The samples were photographed, and the numbers of visible colonies were counted.

### 2.6. Tumor Xenograft Model in Nude Mice

Differently treated cells were subcutaneously injected into the back of male BALB/c nude mice (Vital River Laboratory Animal Technology, Beijing, China). Tumor volumes were measured twice per week. The effects of pristimerin, pristimerin+control siRNA, or pristimerin+CDKN1B siRNA on tumor growth were determined. Twenty-four days after implantation, mice were sacrificed, and tumors were dissected.

### 2.7. Electron Microscopy (EM)

Cells were treated as above. The cells were harvested, washed, and fixed overnight with 2.5% glutaraldehyde (G6257; Sigma-Aldrich, St. Louis, MO) containing 1% tannic acid. After washing, the cell pellets were embedded in Araldite-Epon. The ultrathin sections were observed with a Hitachi h-7650 electron microscope, and representative images were analyzed.

### 2.8. Data Analysis

Data were obtained from at least three independent experiments and presented as the mean ± SEM. We used a two-tailed, unpaired Student's *t*-test for all pairwise comparisons (GraphPad Prism version 5). *P* < 0.05 was considered to represent a significant difference.

## 3. Results

### 3.1. Pristimerin Inhibits Human Esophageal Cancer Cell Proliferation

**Figure 1:**
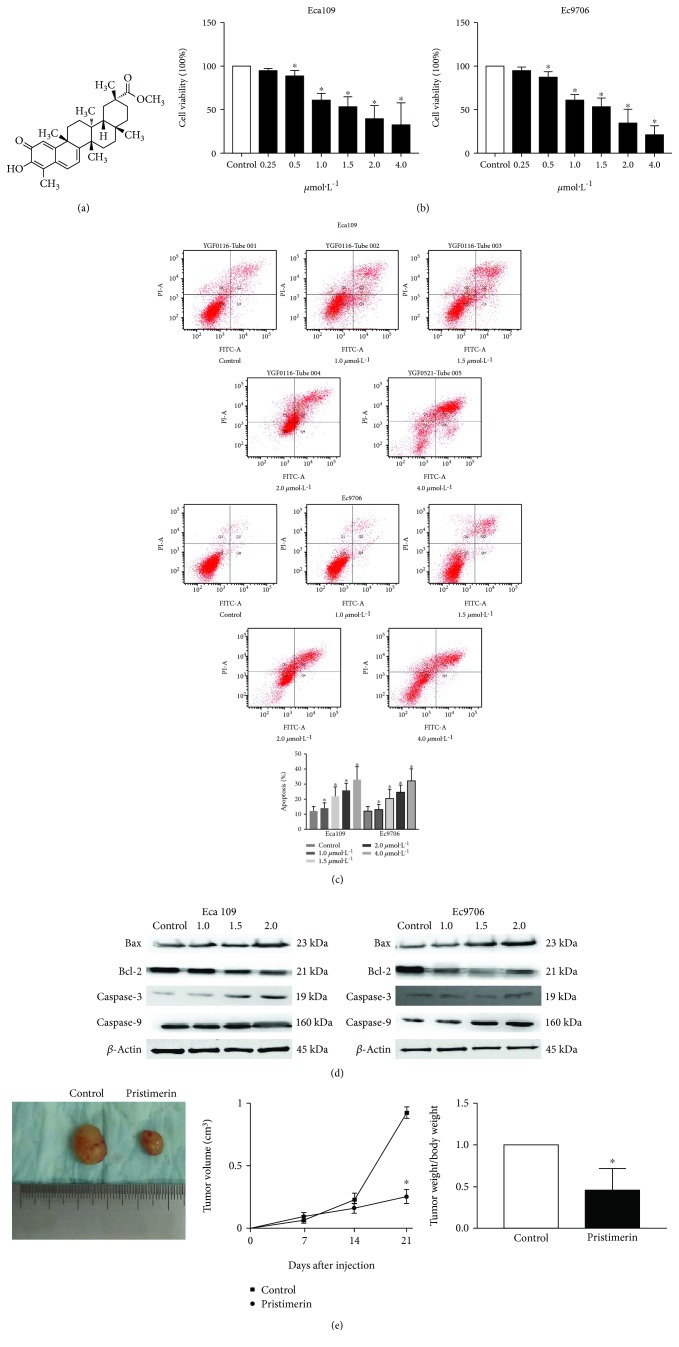
Pristimerin inhibits human esophageal cancer cell proliferation. (a) Molecular formula of pristimerin. (b) Eca109 and Ec9706 cells were treated with pristimerin at concentrations of 0.5, 1.0, 1.5, 2.0, and 4.0 *μ*mol·L^–1^ for 48 h. Relative cell viability determined by the CCK-8 assay. (c) Cytometric analysis of Eca109 and Ec9706 cells treated with pristimerin (0.5, 1.0, 1.5, 2.0, and 4.0 *μ*mol·L^–1^, 48 hours). (d) Protein level of Bcl-2, Bax, caspase-3, and caspase-9. (e) Effect of pristimerin on the growth of Eca109 cells injected into nude mice. Male BALB/c nude mice were subcutaneously injected with 5 × 10^6^ Eca109 cells infected with pristimerin. Tumor volume and weight were monitored over time as indicated, and the tumor was excised and weighed after 21 days. ^∗^*P* < 0.05 versus control group. *n* = 3 independent experiments for each group.

To investigate the role of pristimerin (Figure 1(a)) in esophageal cancer cells, the antiproliferative effect of pristimerin on Eca109 and Ec9706 cells was examined by exposing the cells to different concentrations (0.5, 1.0, 1.5, 2.0, and 4.0 *μ*mol·L^–1^) for 48 h (Figure 1(b)).

Flow cytometric analysis showed that pristimerin treatment induced significant apoptosis in Eca109 and Ec9706 cells in a dose-dependent manner (Figure 1(c)). In the presence of 1.5 *μ*mol·L^–1^ pristimerin, proliferation of Eca109 and Ec9706 cells was inhibited by approximately 50% after treatment for 48 h. This concentration and treatment time period were therefore used in subsequent experiments. We also investigated apoptosis-related proteins. We found that Bcl-2 was downregulated, whereas caspase-3, caspase-9, and Bax were upregulated by pristimerin in a dose-dependent manner (Figure 1(d)). These results showed that pristimerin can induce apoptosis in Eca109 and Ec9706 cells.

The xenograft model of human esophageal cancer Eca109 cells in nude mice was used to explore the role of pristimerin (1.5 *μ*mol·L^–1^, 48 h) in tumor proliferation *in vivo*. Eca109 cells transfected with pristimerin were injected subcutaneously into the flank of each nude mouse. The tumor size was measured every 3 days, and growth was plotted against the average tumor size. After 3 weeks, all the mice were sacrificed, and their bodies and the xenografts were weighed. As expected, tumor size and weight in the control group were significantly increased compared to the pristimerin-treated group (Figure 1(e)).

### 3.2. Pristimerin Induces G0/G1 Phase Arrest by Regulating Cell Cycle-Regulated Proteins

**Figure 2:**
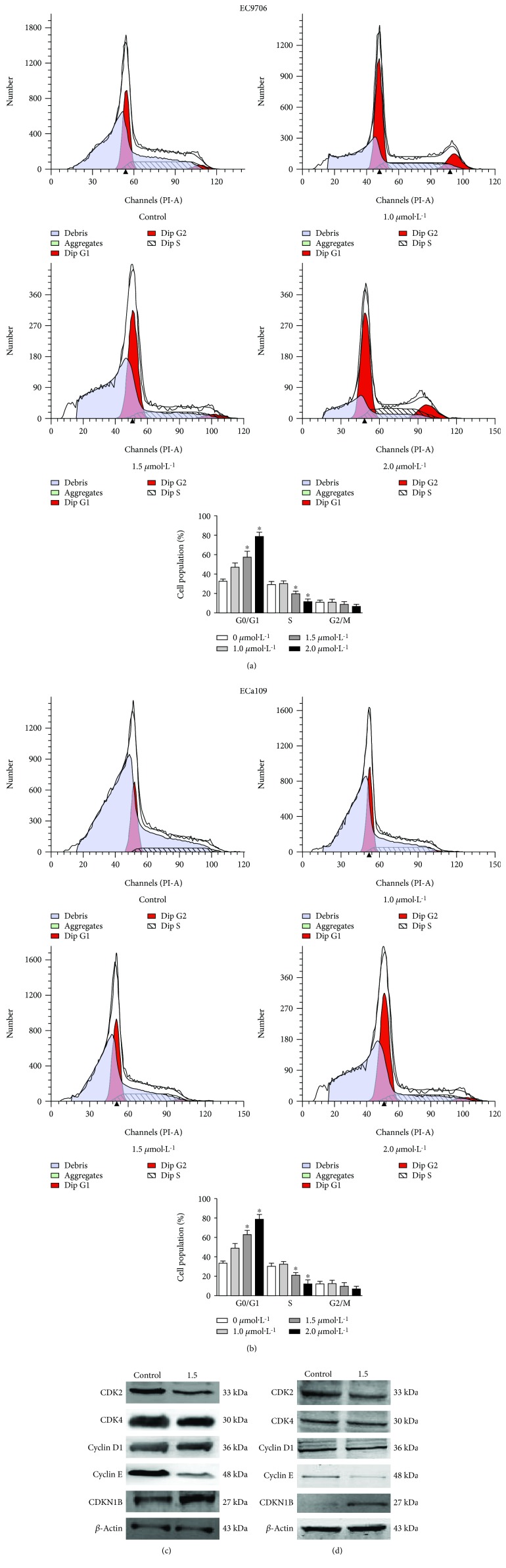
Eca109 and Ec9706 cells were exposed to various concentrations of pristimerin (0, 1.0, 1.5, and 4.0 *μ*mol·L^–1^) for 48 hours followed by the cell cycle distribution assay and altered CDK2, CDK4, cyclin E, and CDKN1B. The percentage of cells in G0/G1, S, and G2/M phases of the cell cycle following pristimerin treatment in Eca109 and Ec9706 cells (a, b). Western blotting was used to detect the ratios of CDK2, CDK4, cyclin E, and CDKN1B expression in Eca109 (c) and Ec9706 (d) cells treated with pristimerin (1.5 *μ*mol·L^–1^, 48 hours). Relative expression of CDK2, CDK4, cyclin E, and CDKN1B was normalized to *β*-actin. Data are the average of three independent experiments for each group. Comparable results were observed from another three experiments. ^∗^*P* < 0.05 versus control group.

To determine whether pristimerin inhibits cell proliferation by inducing cell cycle arrest, we examined the cell cycle distribution in cells treated with pristimerin. As shown in Figure 2, pristimerin led to the accumulation of cells in the G0/G1 phase and a corresponding decrease in G2/M and S phases in both Eca109 (Figure 2(a)) and Ec9706 (Figure 2(b)) cells. To elucidate the mechanisms of action, we measured the expressions of cell cycle-regulated proteins. Pristimerin downregulated the expressions of CDK2, CDK4, and cycle E and upregulated the levels of CDKN1B (Figures 2(c) and 2(d)). These data suggest that pristimerin induces G0/G1 phase arrest by altering the key molecules of G0/G1 cell cycle regulator markers.

### 3.3. Pristimerin Induces Autophagy and Autophagy-Related Protein in Eca109 Cells

**Figure 3:**
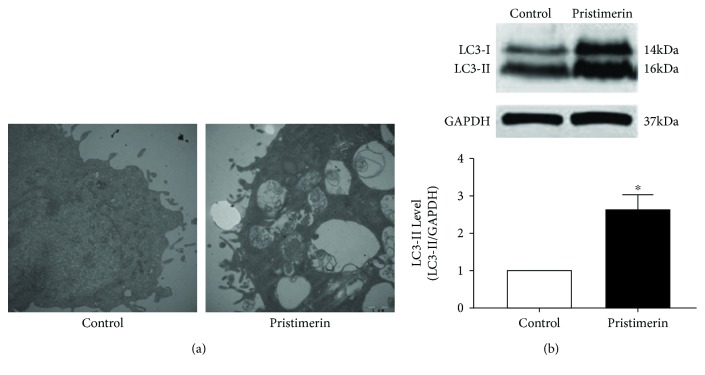
Pristimerin induces expression of autophagy-related protein in Eca109 cells: (a) EM staining of pristimerin-induced autophagy and (b) the protein level of LC3-I/LC3-II. Data are expressed as mean ± SEM; *n* = 3 for each group. ^∗^*P* < 0.05 versus control group.

To investigate the role of pristimerin in autophagy by EM (Figure 3(a)), we further examined the expression of autophagy-related protein LC3-II/LC3-I. The Western blot assay showed that the ratio of LC3-II/LC3-I was significantly augmented after treatment with pristimerin compared with the control group (Figure 3(b)).

### 3.4. Downregulation of CDKN1B Promotes the Growth of Tumors Induced by Pristimerin Inhibition

**Figure 4:**
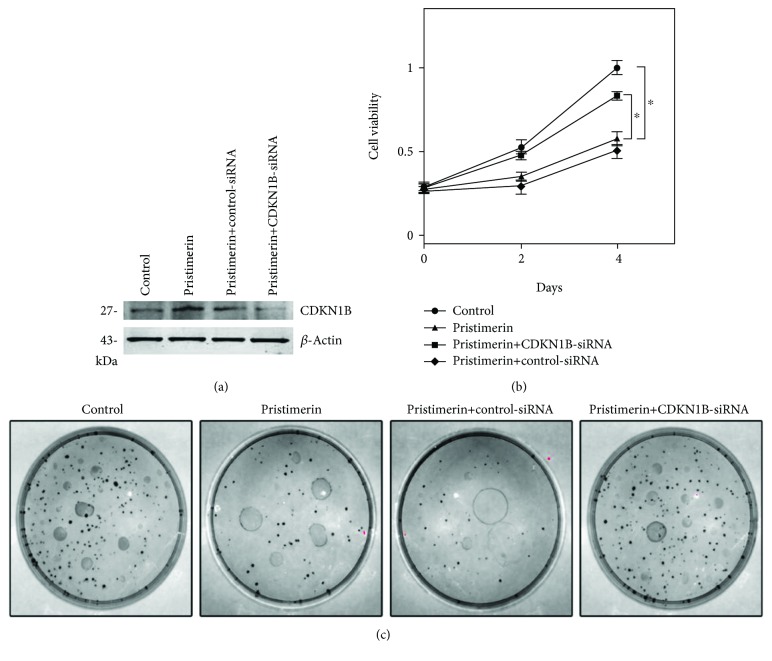
Enforced CDKN1B expression partially reversed the effects of pristimerin in Eca109 cells. (a) Cotransfection of the CDKN1B vector successfully restored CDKN1B expression in Eca109 cells. (b) Exogenous expression of CDKN1B reversed Eca109 cell viability inhibition induced by pristimerin. (c) Exogenous expression of CDKN1B reversed colony formation capability reduction induced by pristimerin. ^∗^*P* < 0.05 versus control group (*n* = 3 independent experiments for each group).

We confirmed that CDKN1B expression was restored by CDKN1B expression vector cotransfection (Figure 4(a)). We found that the viability of Eca109 cells inhibited by pristimerin was reversed as determined by CCK-8 and clonogenic assays (Figures 4(b) and 4(c)).

## 4. Discussion

Accumulating evidence has demonstrated the role of Chinese traditional medicine in preventing tumorigenesis and progression of esophageal cancer. Pristimerin has been found to be a potential novel therapeutic agent for esophageal cancer [15–19]. In the present study, we initially demonstrated that pristimerin induced apoptosis, cell cycle arrest, and autophagy both *in vitro* and *in vivo* in esophageal cancer. Additionally, restoration of pristimerin significantly inhibited cell proliferation in esophageal cancer cell lines by acting on CDKN1B. These findings indicate an anticancer effect of pristimerin in esophageal cancer. Our team have investigated the effect of dihydroartemisinin (DHA) on esophageal cancer cells [13]. We found that DHA could induce apoptosis, cell cycle arrest, and autophagy in esophageal cancer cells. But in our present work, we investigated the anticancer effect of pristimerin and paid more attention on the role of CDKN1B in the mechanism. In addition, we explored the role of CDKN1B *in vitro and in vivo*.

Apoptosis, or the programmed cell death, plays the crucial role in many biological processes of all the diseases. Bcl-2 family is one of the most important regulatory families in the progress of apoptosis. The major function of the Bcl-2 family is to mediate the permeabilization of the outer mitochondrial membrane, which is the most important event in the intrinsic apoptotic pathway [20]. The Bcl-2 family can be divided into antiapoptotic and proapoptotic proteins according to their different structures and function, and all family members possess the highly-conserved BCL-2 homology (BH) domains [21]. The antiapoptotic Bcl-2 family group includes Bcl-2, Bcl-xL, and Bcl-w. The Bcl-xL and Bcl-2 help proteins to localize in the outer mitochondrial membrane by their carboxyterminal hydrophobic transmembrane domain [22]. The cytotoxic signals provoke the Bcl-w associate with the membrane by its conformational changes [23]. The Bax, Bak, and Bok belong to the proapoptotic Bcl-2 family that share three BH domains [24]. By reconstitution of Bak into Bax/Bak, double knockout cells indicate that the Bax might be involved in the outer membrane degeneration, and Bak participate in the early stage of mitochondrial fragmentation [25, 26]. As previously reported, pristimerin significantly induces esophageal cancer cell death through the NF-*κ*B pathway [27]. In contrast, in the present study, we observed a significant decrease in the level of Bcl-2 accompanied by increased levels of Bax, caspase-3, and caspase-9, indicating that pristimerin is able to trigger intrinsic apoptosis thereby inducing esophageal cancer cell death. Similar to our results, previous studies have also demonstrated that pristimerin induces apoptosis in various types of cancer cells, such as breast, colon, and colorectal cancers [28–30].

In addition, previous research indicated that pristimerin could induce G0/G1 phase arrest [27]. Cyclin-CDK heterodimers regulated cell cycle progression through each phase and is fundamental to cell cycle checkpoints in cells. Deregulation of cell-cycle control due to aberrant CDK activity is widespread in most cancer types [31]. In addition, cyclin E, CDK2, and CDK4 are necessary factors involved in the entry into and progression of cells through the G1 phase of the cell cycle. In conclusion, based upon our date, we found that pristimerin could induce the downregulation of cyclin E, CDK2, and CDK4 to lead to the block of G0/G1 transition, which gave rise to cell death in esophageal cancer.

Autophagy is initiated when cells need to produce intracellular nutrients and energy, resulting in cells either undergoing architectural remodeling or eliminating damaging cytoplasmic components [32–36]. Here, our microscopy images showed the activation of autophagy, supported by the elevated accumulation of autophagosome, increased by pristimerin. As previously reported, LC3-I/LC3-II is inversely correlated with selective autophagic protein in many diseases. In the present study, we found that pristimerin increased the ratio of LC3-II/LC3-I. These results indicate that pristimerin may activate the autophagy signaling pathway. Lately, evidence indicated that autophagy plays an important role in tumor initiation and progress. However, a much-debated question is whether autophagy in cancer cells causes death or protects cells [37]. Some authors maintained that autophagy is a protein degradation system in which cellular proteins and organelles are sequestered, delivered to lysosomes. According to other authors, autophagy might be a response to recycle injured organelles to avoid apoptosis [38]. They thought that it actually is an attempt to support survival in response to cellular stress conditions. We found that pristimerin induced apoptosis and autophagy in human esophageal cancer cells. Therefore, there were still many unanswered questions about the effect of autophagy in cancer cells treated by pristimerin and the detailed mechanism between apoptosis and autophagy.

CDKN1B, also known as p27, was initially discovered as a nuclear cell cycle inhibitory protein. p27 exported to the cytoplasm [39, 40] was considered as a mechanism to inactivate the cell cycle inhibitory effects of p27 in the nucleus and to allow human cancer cells to escape cell cycle arrest [41–43]. However, in the present study, we found that treatment with pristimerin significantly upregulated p27 expression. Previously, Jia showed that autophagy regulates T lymphocyte proliferation through selective degradation of the cell cycle inhibitor CDKN1B/p27^Kip1^ [44]. Chen et al. suggested that p27 protein protects metabolically stressed cardiomyocytes from apoptosis by promoting autophagy [45]. Here, we showed that knockdown of CDKN1B can inhibit esophageal cancer cell growth.

In conclusion, our results demonstrate that pristimerin induces apoptosis, cell cycle arrest, and autophagy in esophageal cancer cells. In addition, downregulation of CDKN1B promotes the growth of tumors induced by pristimerin inhibition. These findings may improve the understanding of the role of pristimerin in esophageal cancer and may provide a potential therapeutic agent for this cancer.

## Data Availability

The data of the cell viability assay, clonogenic survival assay, cell apoptosis and cycle analysis, electron microscopy, and Western blotting and the data in vivo used to support the findings of this study are included within the article. And no data used to support the findings of this study are included within the supplementary information file.
